# Evolutionary dynamics and molecular adaptation of Rift Valley fever virus across human and non-human outbreaks in Africa

**DOI:** 10.1186/s12864-026-13002-4

**Published:** 2026-06-15

**Authors:** Isaac Emmanuel Omara, John Juma, Derek Tshiabuila, Graeme Dor, Eduan Wilkinson, Cheryl Baxter, Houriiyah Tegally, Monika Moir, Samuel O. Oyola, Tulio de Oliveira

**Affiliations:** 1 Centre for Epidemic Response and Innovation (CERI), School for Data Science and Computational Thinking, Stellenbosch University, Stellenbosch, South Africa; 2 International Livestock Research Institute (ILRI), Old Naivasha Road, Nairobi, Kenya; 3 Division of Medical Virology, Faculty of Medicine and Health Sciences, Stellenbosch University, Stellenbosch, South Africa; 4 Centre for the AIDS Programme of Research in South Africa (CAPRISA), Durban, South Africa; 5 KwaZulu-Natal Research and Innovation Sequencing Platform (KRISP), University of KwaZulu-Natal, Durban, South Africa

**Keywords:** Rift Valley fever virus, Evolutionary dynamics, Adaptive evolution, Diversifying selection, Genomic surveillance, Phylogenetics, Africa Outbreaks

## Abstract

**Background:**

Rift Valley fever virus (RVFV) is an emerging zoonotic virus of major public health and veterinary concern across Africa. Although past genomic studies have focused on outbreak response and lineage classification, the molecular mechanisms driving viral persistence and adaptation remain poorly understood. This study aimed to identify RVFV protein-coding mutations and adaptive signatures across multiple epidemics and epizootics in Africa.

**Methods:**

This retrospective genomic analysis examined 596 RVFV segment sequences retrieved from the NCBI Virus database, including L (*n* = 173), M (*n* = 196) and S (*n* = 227) sequences from 13 African countries across human and non-human hosts between 1944 and 2022. Following the identification of protein-coding mutations via Genome Detective and custom scripts, we performed phylogenetic reconstruction using IQ-TREE and host-state reconstruction to map cross-species transmission patterns and selection pressure analyses were conducted using codon-based models implemented in the Datamonkey platform. All data analyses and visualizations were performed using R software.

**Results:**

A total of 7,339 protein-coding mutations were identified, ranging from 2—20 per isolate. RVFV isolates collected in South Africa, Kenya and Madagascar exhibited the highest genomic diversity. Comparative analysis revealed higher mutation burdens in the L and S segments than in the M segment, with broader diversity among non-human hosts. Phylogenetic reconstruction showed that human-derived sequences clustered within livestock and vector lineages, a pattern consistent with significant genetic bottlenecks during spillovers. Notably, host-state reconstruction identified livestock lineages as the primary source of human outbreaks. We identified seven recurrent amino acid mutations across the genome: N277S, N277D and S278N in the polymerase (L); I442S, I442V, V659A in the glycoproteins (M); and N133S in the NSs protein (S). FUBAR-supported signals consistent with diversifying selection were identified at corresponding codon sites, particularly within the polymerase and glycoprotein regions, highlighting candidate residues potentially associated with adaptive processes affecting replication efficiency and immune evasion.

**Conclusion:**

Our findings demonstrate that RVFV evolution across Africa is geographically and temporally heterogeneous, with livestock infections identified as the primary driver of human outbreaks. While RVFV evolution is largely shaped by purifying selection, FUBAR analysis revealed a limited number of candidate codon sites under diversifying selection that may facilitate host-specific adaptation. These host-specific pressures likely contribute to adaptive substitutions that fine-tune polymerase function, alter glycoprotein antigenicity and enhance immune escape. Collectively, these results reveal the molecular mechanisms underpinning viral persistence and provide information to support the design of cross-protective vaccines.

**Supplementary Information:**

The online version contains supplementary material available at 10.1186/s12864-026-13002-4.

## Background

Rift Valley fever virus (RVFV) is an emerging zoonotic virus of major public health and veterinary importance across Africa and the Arabian Peninsula [1]. The virus belongs to the genus *Phlebovirus* in the family *Phenuiviridae* [2] and is responsible for the Rift Valley fever (RVF). RVFV is a tripartite, negative sense RNA virus consisting of large (L), medium (M) and small (S) segments [3]. The L segment encodes the RNA-dependent RNA polymerase necessary for viral replication, the M segment encodes envelope glycoproteins Gn and Gc along with non-structural proteins (NSm), while the S segment uses an ambisense strategy to encode the nucleocapsid protein (N) and the non-structural protein NSs [4]. These structural and non-structural proteins play central roles in viral replication, pathogenesis and immune evasion [5]. Mutations within these genomic regions, particularly in the glycoproteins and NSs protein, have been linked to altered virulence, host range adaptation and immune suppression, making them critical targets for genomic and immunological studies [6]. Notably, the NSs protein is a major virulence factor known to antagonize host interferon responses, while the Gn and Gc glycoproteins facilitate viral entry and immune recognition [7].

Since the discovery of RVF in the Great Rift Valley region in Kenya in 1930 [8], Africa has experienced over thirty different RVFV outbreaks in both humans and livestock, leading to substantial economic losses, morbidity and mortality [1]. Countries in Africa with reported RVFV outbreaks include; Egypt [9], Sudan [10], Somalia [11], Kenya [12], Uganda [13], Tanzania [14], Rwanda [15], Burundi [16], Madagascar [17], Mauritania [18], Niger [18], Senegal [19], Central African Republic [20], Namibia [21] and South Africa [4]. RVFV transmission occurs primarily through mosquito vectors such as species of the *Aedes* and *Culex* genera [8], but direct contact with infected animal tissues or fluids also contributes to spillover events in humans [5]. RVFV infection in animals, particularly in ruminants may lead to abortion storms and high neonatal mortality [1]. In human, RVFV infections may result in hemorrhagic fever, encephalitis and sometimes death [6]. Given the broad host range, complex transmission dynamics and its potential for geographic spread, RVFV has been designated as a high-priority pathogen by the World Health Organization, the Vaccines Alliance (GAVI) [19] and Coalition for Epidemic Preparedness Innovations (CEPI) [22].

While several genomic and evolutionary studies of RVFV have focused on outbreak response through lineage classification and phylogeographic spread [23], less attention has been paid to the molecular processes that underpin RVFV adaptation and persistence. Particularly, it’s mutation diversity, accumulated over decades of outbreaks and selection pressures that shape its persistence and cross-species transmission in Africa [1]. Like many RNA viruses, RVFV, exhibits a high mutation rate that leads to extensive genetic diversity, enabling rapid adaptation to shifting ecological and host environments [24]. This genetic adaptability may alter replication efficiency, influence viral fitness, immune evasion and the capacity to cross species barriers [25]. Identifying which of these mutations are positively selected and contribute to adaptive evolution is essential not only for understanding viral emergence risks [26] but also for identifying stable targets for vaccine design [24].

Although the increasing availability of RVFV whole genomes in public repositories [23] has enabled investigations of its molecular evolution [27], most existing studies to date still focus on lineage diversity through phylogenetic classification [21], leaving a fragmented view of how viral diversity and adaptation unfold across African outbreaks. This limited focus has partly been due to sparse genomic surveillance [28], uneven geographic sampling and lack of integrated analytical frameworks that jointly identify mutations, determine selection pressures and host-associated evolutionary dynamics [29]. As a result, the evolutionary patterns and adaptive features that enable RVFV to persist, adapt across different hosts and evade immune responses remain poorly understood [1]. This knowledge gap limits our ability to predict emergence risks and hinders the design of vaccines that target evolutionarily stable epitopes. Here, we address this gap by conducting the first continent-wide comparative analysis to identify protein coding mutations and codon level selection pressures across both human and non-human isolates. We analyzed RVFV genetic diversity across the L, M and S genome segments from multiple outbreaks since 1944. By mapping mutations to signals of adaptive evolution, these findings provide new insights into the evolutionary pressures that shape RVFV persistence and adaptation during outbreaks in Africa.

## Methods

### Study design

This was a retrospective genomic study utilizing 596 RVFV segment sequences retrieved from the National Centre for Biotechnology Information (NCBI) virus database [23]. Using the NCBI virus database search query, sequences were retrieved using the taxonomy identifier: Rift Valley fever virus (taxid:11,588). Filters were applied to retain nucleotide sequences referred to as “complete”, restricted to “African countries” and belonging to either L, M or S segments. Sequences were excluded if they were partial or if they contained > 5% ambiguous nucleotide characters. Duplicate sequences were removed and other records annotated as vaccine strains or lab- passaged isolates were excluded to retain naturally circulating virus strains. These filtering steps were to ensure high alignment quality both at the nucleotide and amino acid levels ensuring codon-correct sequences.

### Identification of protein-coding mutations and lineage assignment

Protein-coding mutations in the RVFV M segment were identified using the viral identification tool, Genome Detective [30]. This tool identifies protein-coding mutations and performs phylogenetic characterization by aligning viral sequences to the M segment reference genome (NC_014396.1) using DIAMOND and Advanced Genome Aligner. All sequences from the M segment were uploaded as a multi FASTA file to the platform and classification was performed by sequence similarity and lineage assignment. A custom Python script was used to extract all the protein-coding mutations from the generated XML file.

Since the Genome detective tool performs mutation identification and lineage assignment primarily for the RVFV M segment, mutation classification for the L and S genome segments, was performed using a Nextflow v23.04.4 [31] workflow implemented in the pipeline by Juma et al. [32]. This workflow aligned sequences to the L segment reference genome (NC_014397.1) and S segment reference genome (NC_014395.1), identified amino acid changes and assigned lineages based on RVFV phylogenetic definitions provided in the pipeline. Input reformatting and metadata harmonization were done using R software, alignment of viral sequences performed using MAFFT (version 7.505) [33], followed by manual trimming with Aliview (version 1.28) [34]. Duplicate sequences were removed using seqkit (version 2.6.1) [35].

The nucleotide sequences were translated to amino acids using Biopython (version 1.83) [36] enabling identification of protein-coding mutations. The Single Nucleotide Polymorphisms (SNPs) across the aligned RVFV genome segments were identified relative to their corresponding reference genomes and visualized using SniPIT [37]. SNP visualization was used to confirm nucleotide-level variation underlying recurrent amino acid substitutions across the L, M and S genome segments. All amino acid mutations were extracted, tabulated and aggregated by country, accession, lineage, year of sample collection and host (human, livestock, wildlife and vector). The resulting dataset was imported into R (version 4.4.3) [38] for computation of descriptive statistics which included, mean, median, standard deviation and interquartile range, all of which were computed to summarize protein-coding mutation counts per isolate by country, genome segment and host category. The difference in mutation counts between countries was assessed using the non-parametric Kruskal–Wallis test [39], while differences in mutation burdens between human and non-human host groups within each segment were assessed using the Wilcoxon rank-sum test, given the non-normal distribution of mutation counts [40]. The two-sided p-values (*p* < 0.05) were considered statistically significant. The median differences and 95% confidence intervals were reported.

### Recombination detection

The amino acid (codon) alignment for each of the RVFV segment sequences (S, M, L) was performed using MAFFT (version 7.505) [33] and uploaded into the Datamonkey web-based server [41] and screened for recombination breakpoints using the Genetic Algorithm for Recombination Detection (GARD) implemented in the server [42]. There was no statistically significant evidence of recombination detected in all three fragments (ΔAIC-c ≈ 0) [43].

### Phylogenetic analysis

Maximum likelihood phylogenetic trees were reconstructed for all three genomic RVFV segments. Multiple sequence alignments were generated with MAFFT(version 7.505) [33] and trimmed using trimAl (version 1.4.1) [44] to remove poorly aligned regions. Maximum likelihood phylogenies were inferred using IQ-TREE (version 2.3.5) with the best fit substitution model selected by ModelFinder and branch support determined using 1000 ultrafast bootstrap replicates (-B 1000) and 1000 SH-aLRT tests [45]. To assess the temporal signal, regression of root-to-tip genetic distance against sampling date (years) was performed using TreeTime (version 0.11.4) [46]. Temporal outliers by more than three standard deviations were automatically filtered using the –clock-filter 3 option. Time-scaled phylogenetic trees were then reconstructed using TreeTime (version 0.9.4) [47], incorporating sampling dates from the metadata. The visualization and annotation of host, region and lineage clustering were performed in R (version 4.4.3) using ggtree (version 3.10.1) and ggtreeExtra (version 1.12.1) packages.

### Host-state reconstruction and spillover analysis

To infer host transmission patterns of RVFV, ancestral host states were reconstructed along the time-scaled RVFV M segment phylogenetic tree. M was selected as a representative segment for host-state analysis because it encodes the envelope glycoproteins, which play key roles in host interaction, viral entry and immune recognition [48]. The tree was rooted at the midpoint and multichotomies were resolved prior to analysis [49]. Sequence metadata were harmonized to include discrete host categories (human, livestock, wildlife and vector) and the phylogeny was pruned to retain only tips with corresponding host information.

The ancestral host states were inferred in R using the ace() function implemented in ape package [49], applying a discrete equal rates (ER) Markov model. For each internal node, the most probable host state was assigned based on the maximum likelihood estimate of the ancestral character states. Host to host transitions were identified by comparing inferred host states between parent and child nodes along each branch of the phylogeny generating a directed host transition matrix that summarizes evolutionary transitions across the tree [50].

Spillover events into humans were defined as transitions in which the inferred ancestral state corresponded to a non-human host and the descendant node corresponded to a human state. The transition counts were summarized to quantify the magnitude and directionality of cross host transmission across the evolutionary history of RVFV [51]. The resulting transition matrix was visualized as a directed network using the igraph package [52], with nodes representing host categories and edges weighted according to the frequency of inferred transitions. All the analyses were performed using R (version 4.4.3) software [38].

### Selection pressure analysis

Signatures of selection pressure were detected using the Datamonkey web-based platform [53] implemented in HyPhy (version 2.5.63). Codon-aligned FASTA files of the coding sequences from the L, M and S genome segments were uploaded to the data monkey server and three complementary methods were applied to detect codons under diversifying selection: FUBAR (Fast Unconstrained Bayesian Approximation) [54], SLAC (Single Likelihood Ancestor Counting) [55], and FEL (Fixed Effects Likelihood) [56]. Codons were considered to show evidence of diversifying selection if they met significance thresholds of *p* ≤ 0.1 for SLAC and FEL [57] or posterior probability ≥ 0.9 for FUBAR [54].

The outputs from these analyses were grouped by host (humans vs non-human) and by genome segment (L, M and S) to assess host-specific evolutionary pressures. These models provide a robust evaluation of codon-level selection, allowing both strong and weak patterns of adaptive evolution to be detected across the L, M and S RVFV genome segments. The dN/dS (β/α) ratio compares the rate of nonsynonymous to synonymous substitutions and was used to infer evolutionary pressure. Sites were therefore considered significant at posterior values ≥ 0.90 based on FUBAR outputs [54].

## Results

### Dataset composition and genomic coverage

Of the 659 RVFV segment sequences available in the NCBI virus database at the time of retrieval (July 2025), 596 complete RVFV segment sequences met the inclusion and exclusion criteria. These records represent pooled complete RVFV segment sequences across the three RVFV segments, which are L (*n* = 173), M (*n* = 196) and S (*n* = 227), from 13 African countries across human and non-human hosts between 1944 and 2022. This dataset was used to identify protein-coding mutations, determine the selection pressure and perform ancestral host-state reconstruction.

### Spatiotemporal distribution of RVFV protein-coding mutations across Africa

**Fig. 1:**
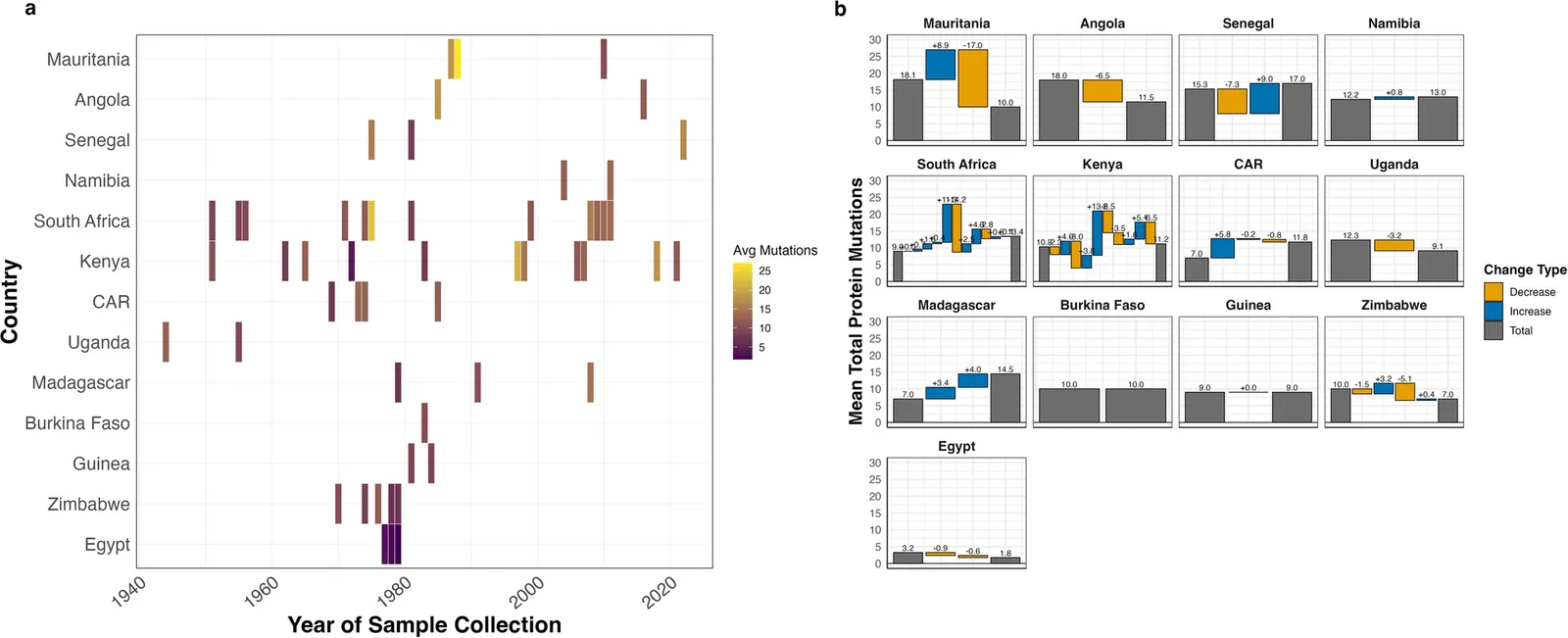
Spatiotemporal distribution of RVFV protein-coding mutations in Africa. **a** Mean protein-coding mutation count per isolate by country and year aggregated across L, M and S genome segments. **b** Stepwise changes in mean mutation burden between consecutive sampled years for each country. Gray bars represent the mean total protein-coding mutations for each sampled year. Two gray bars are shown for each comparison because they represent the paired adjacent timepoints used to calculate the change between consecutive sampled years. Blue bars indicate increases and yellow bars show decreases in mean mutation burden between consecutive sampled years

Across the 596 RVFV segment sequences analyzed, 7,339 protein-coding mutations were identified (Dataset S4). The mutation burdens varied across geography and time, ranging from 0 to > 20 mutations per isolate (Fig. 1a). Countries such as Mauritania during the 1987 outbreak (*n* = 8, mean = 18.1 mutations per isolate, range = 8–28) and South Africa during the 2008–2011 outbreaks (*n* = 290, mean = 13.4 range = 8–31) showed the highest mean protein mutation counts. These show periods in which isolates accumulated more amino acid changes relative to other times and locations. In contrast, Egyptian isolates from the 1970 s showed low protein mutation counts (*n* = 16, mean = 2.4, range = 1–5). Sporadic increases in protein mutation counts were also evident in Senegal (*n* = 5, mean = 14.2, range = 8–25), Angola (*n* = 3, mean = 13.7, range = 11–18) and Zimbabwe (*n* = 27, mean = 8.0, range = 3–17).

The year-to-year changes in mean protein-coding mutations (Fig. 1b) were defined as stepwise differences between consecutive sampled years for each country, where each step represents the change in mean mutation count between two temporally adjacent outbreaks. Kenya showed pronounced variability across nine step transitions with increases of up to + 13.2 mutations between successive sampling intervals and declines of up to −8.0 mutations, reflecting dynamic shifts in mutation burden across multiple outbreak periods. South Africa also demonstrated marked fluctuations across eleven step transitions with an increase of + 11.3 mutations and a sharp decrease of −14.3 mutations during the 2008–2011 outbreak period, consistent with sustained viral circulation and potential lineage turnover.

In contrast, Senegal, Uganda and Egypt maintained relatively stable trajectories with smaller incremental changes. Senegal showed an increase of + 9.0 mutations and a decrease of −7.3 mutations, which is suggestive of episodic rather than sustained diversification. Whereas Uganda showed a single step transition of −3.2 mutations and Egypt in the 1970 s exhibited minimal changes (−0.6 to −0.9 mutations per step), which is likely due to limited genomic sampling.

### Country-level distribution of mutation burden across Africa

**Fig. 2:**
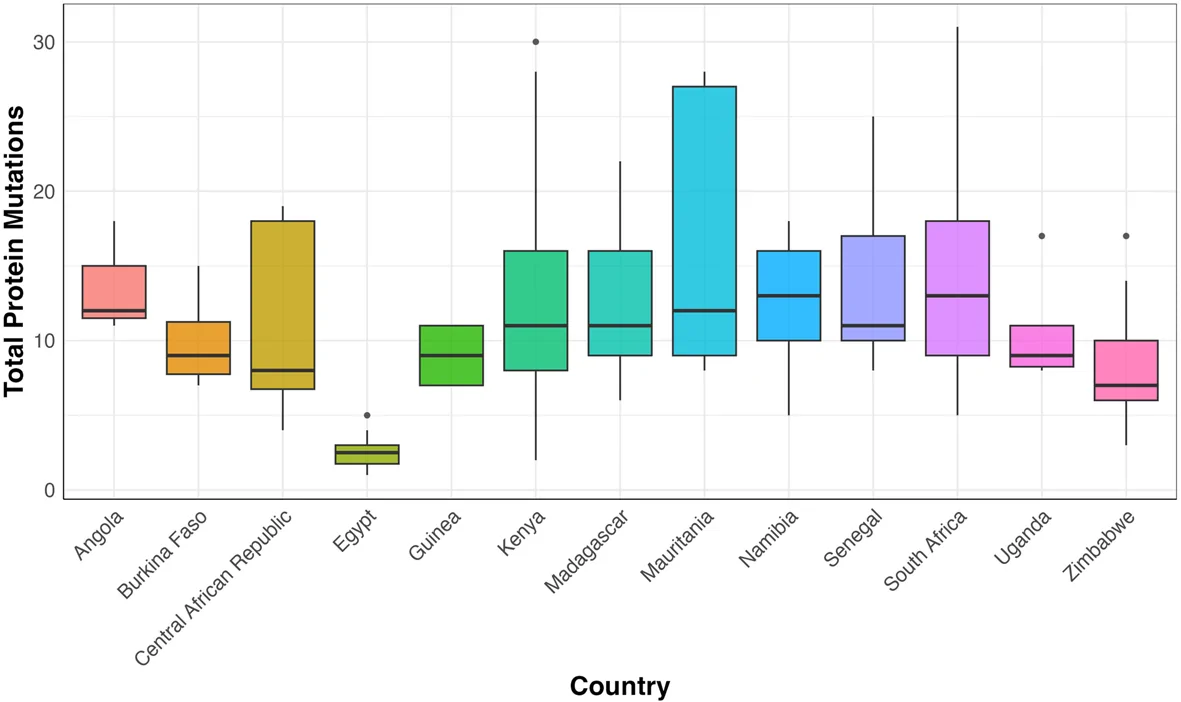
Country-level distribution of mutation burden across Africa. The plot shows the distribution of total protein-coding mutations per isolate. The boxes represent the interquartile range (IQR), horizontal lines indicate medians and the points represent individual RVFV isolates

Mutation frequency varied significantly between individual countries, reflecting the heterogeneous mutation burdens across the African continent. Egypt (*n* = 16) had the lowest frequency (median = 2.5, mean = 2.44, standard deviation = 1.15, inter quartile range (IQR) = 1.75–3.00), while South Africa (*n* = 321) showed one of the highest (median = 13.0, mean = 13.22, standard deviation = 4.30, IQR = 9.0–18.0). Mauritania (*n* = 11) also had elevated values (median = 12.0, mean = 17.45, standard deviation = 9.41, IQR = 9.0–27.0), reflecting greater variability. Namibia (*n* = 7) showed a broad distribution with elevated mutation counts and an extended IQR suggesting within country diversity despite a small sample size. Kenya (*n* = 136) and Madagascar (*n* = 33) both had medians of 11 mutations, but showed wider spreads (Kenya mean = 12.01, standard deviation = 4.84, IQR = 8.0–16.0; Madagascar (mean = 12.91, standard deviation = 4.54, IQR = 9.0–16.0), consistent with greater within country variability (Fig. 2).

### Distribution of protein-coding mutations by segment and host category

**Fig. 3:**
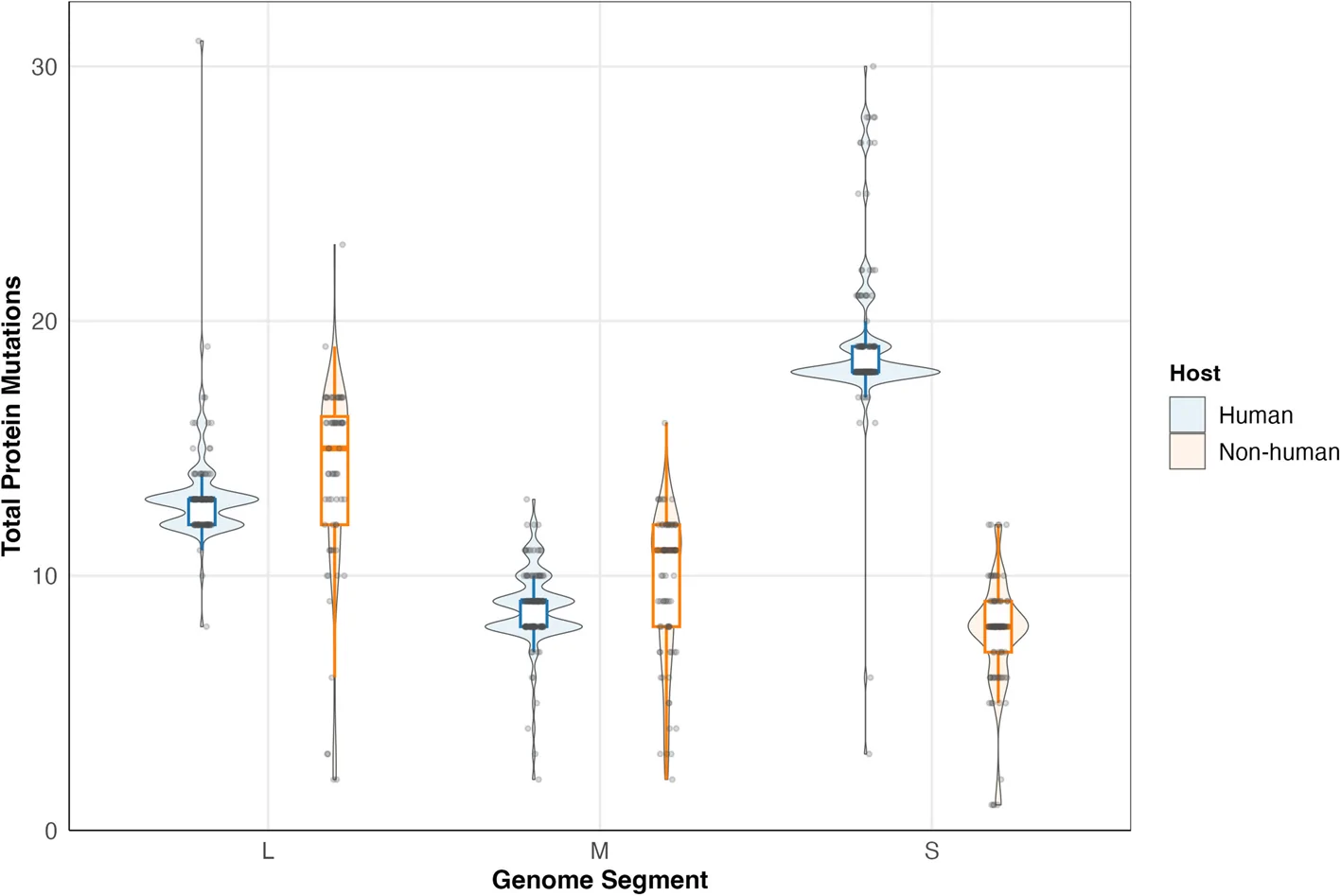
Distribution of protein-coding mutations by segment and host category. The violin plots illustrate the density of total protein-coding amino acid substitutions per isolate relative to the reference sequence. The embedded boxplots indicate the median and interquartile range (IQR). The individual points represent RVFV isolates included in this analysis. Non-human sequences are shown in orange, and the human-derived sequences are shown in blue

The distribution of total protein-coding mutations varied significantly across genome segments and host categories (Fig. 3). In the L segment, non-human isolates (median = 15, IQR = 12.0–16.3; *n* = 68) had significantly higher mutation counts than human isolates (median = 13, IQR = 12.0–13.0; *n* = 105; Wilcoxon rank-sum test, *p* < 0.001; median difference = −2.0, 95% CI: −3.00 to −1.00). Similarly, in the M segment, non-human isolates (median = 11, IQR = 8.0–12.0; *n* = 78) showed higher mutation counts than human isolates (median = 9, IQR = 8.0–9.0; *n* = 118; *p* < 0.001; median difference = −1.0, 95% CI: −2.00 to −1.00). In contrast, the S segment showed markedly higher mutation counts in human isolates (median = 18, IQR = 18.0–19.0;* n* = 131) than in non-human isolates (median = 8, IQR = 7.0–9.0; *n* = 96; *p* < 0.001; median difference = 11.0, 95% CI: 10.00 to 11.00).

### Phylogenetic structure and lineage diversity of the RVFV M segment

**Fig. 4:**
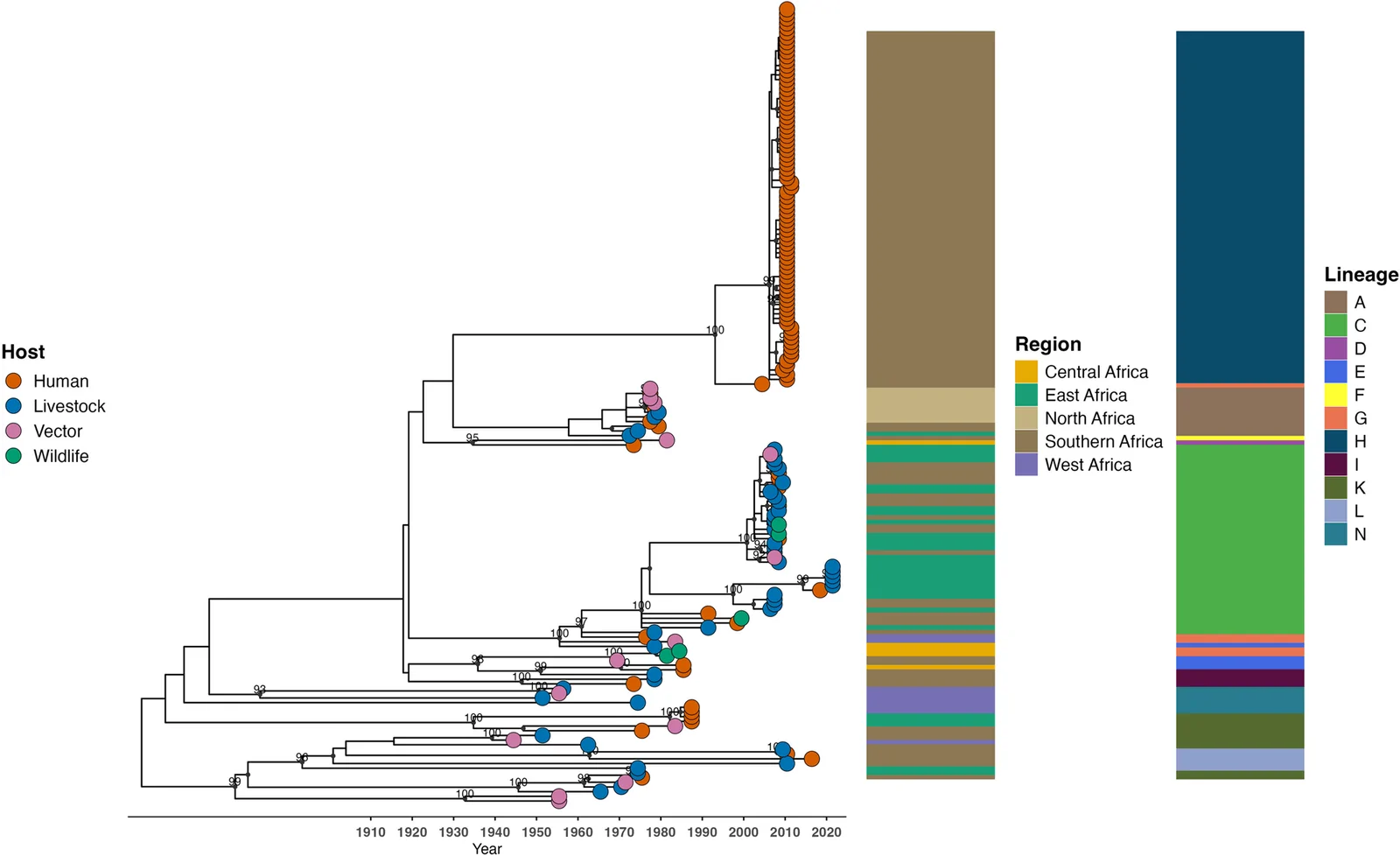
Phylogenetic structure of RVFV M segment by host and geographic region. The tip colors represent host category (Human, Livestock, Wildlife and Vector). The host legend is on the left-hand side. Similar phylogenetic structures are observed for L and S genome segments (Figure S2 and S3). The adjacent heatmaps on the right annotate lineage and geographic regions (North Africa: Egypt, East Africa: Uganda, Kenya, Madagascar, South: Southern Africa, Namibia, Zimbabwe, Central Africa: Central African Republic, Angola and West Africa: Mauritania, Senegal, Burkina Faso, Guinea) and by RVFV lineage. Bootstrap values are shown on major internal nodes to indicate statistical support (≥70%)

The root-to-tip regression analysis of the RVFV M segment demonstrated a positive relationship between sequence divergence and sampling time (years), with an estimated β = 1.88 × 10^–4^ substitutions/site/year (Figure S1), supporting clock-like evolution of the dataset. RVFV lineages showed uneven geographic distributions across the 13 African countries (Fig. 4). Egypt was associated with lineage A consistent with a historically conserved viral population. East African countries including, Uganda, Kenya and Tanzania together with Madagascar showed greater lineage diversity, dominated by lineage C and H, with Kenya additionally harboring lineage I, which was previously detected during the 2006–2007 epidemic [58]. Southern Africa displayed the highest lineage heterogeneity, harboring lineages (H, C, K and L), while Zimbabwe and Namibia were associated with lineages K and H. Sequences from the Central African Republic clustered with lineage D and West African countries showed more restricted lineage profiles. Mauritania, Senegal and Burkina Faso had lineage C, whereas Angola, although represented by fewer sequences was linked to lineage H. Overall, RVFV lineage diversity was unevenly distributed across Africa with higher diversity observed in regions with repeated outbreaks.

### Host-state reconstruction and spillover dynamics

**Fig. 5:**
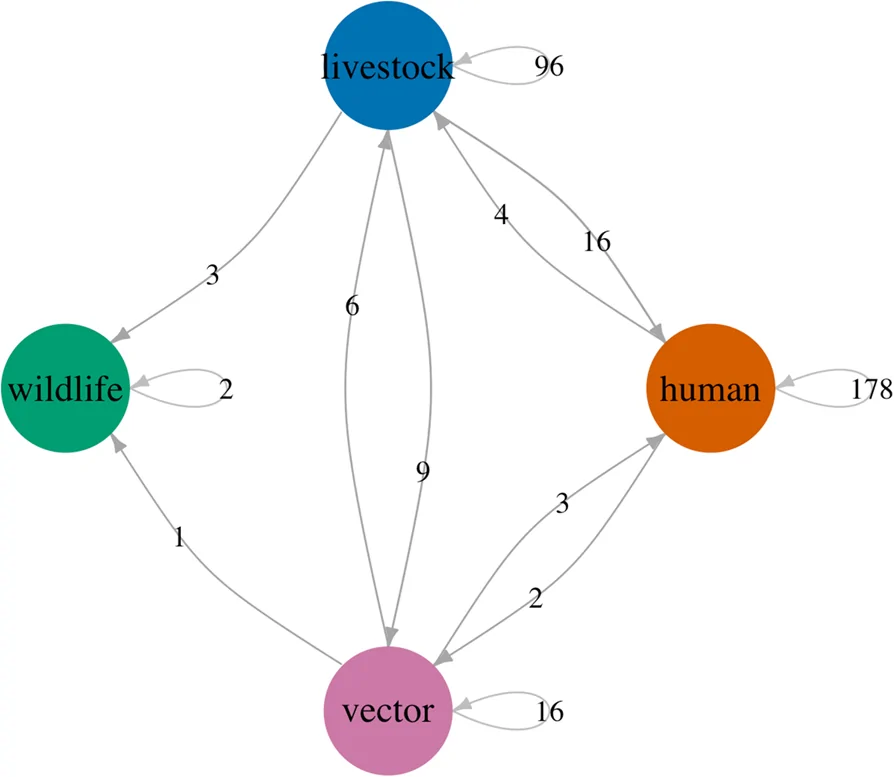
Network of inferred host-to-host transitions for the RVFV M segment. The directed network summarizes ancestral host-state transitions reconstructed along the branches of the time- scaled M segment phylogeny. Nodes represent hosts (human, livestock, wildlife and vector). The directed edges indicate inferred transitions between host states. The thickness in the edge is proportional to the number of inferred transitions, while self-loops represent within host evolutionary transitions. The arrow labels indicate inferred transition counts between host categories. Livestock accounted for the highest number of inferred transitions into humans (*n* = 16), followed by vectors (*n* = 3). There were no direct wildlife-to-human transitions inferred

In addition, host-state reconstruction based on the RVFV M segment phylogeny revealed structured patterns of host-associated evolution across the dataset (Fig. 5). Most inferred transitions leading to human infections originated from livestock lineages (*n* = 16). A small number of inferred transitions into humans arose from vector-associated ancestral nodes (*n* = 3), reflecting phylogenetic host state changes. There was no direct wildlife to human transitions inferred (*n* = 0). These numbers represent the number of inferred host-state transitions along branches of the phylogeny, rather than the number of sampled sequences. The remaining transitions reflected within host evolution including human–human (*n* = 178), livestock-to-livestock (*n* = 96), vector-vector (*n* = 16) and wildlife-to-wildlife (*n* = 2) [59].

### Recurrent mutations associated with viral evolution and host adaptation

After comparative analysis of the L, M and S genome segments, seven nonsynonymous mutations were identified to occur across multiple outbreaks. These mutations occur in functionally important proteins and show evidence of host-specific positive selection [60]. They were selected based on their geographic spread, frequency and detection at codon sites under positive selection in either human or non-human hosts. The mutations were identified in key viral proteins involved in replication (L polymerase), immune recognition and entry (M encoded glycoproteins) [5] and immune suppression (NSs gene) [61]. Table 1 below, summarizes the genomic region, mutations, codon sites, amino acid changes and their inferred biological significance from research studies on these proteins.

**Table 1:** List of recurrent mutations associated with viral evolution and host adaptation

Region	Mutations	Codon Site	Amino acid change	Biological Significance
L	N277S	277	N → S	May alter polymerase function which is linked to host adaptation in humans [62]
	N277D	277	N → D	Introduces negative charge which could affect RNA binding or replication efficiency [63]
	S278N	278	S → N	May change hydrogen bonding and remove regulatory phosphorylation [63]
M	I442S	442	I → S	May affect glycoprotein folding and immune recognition in humans [5]
	I442V	442	I → V	Fine-tunes glycoprotein structure across hosts [5]
	V659A	659	V → A	May adjust glycoprotein packing, positively selected in reservoirs
S	N133S	133	N → S	May disrupt glycosylation motif which could alter immune evasion or protein stability [59]

Interpretation of amino acid changes are based on: A = Alanine, C = Cysteine, D = Aspartic acid, E = Glutamic acid, F = Phenylalanine, G = Glycine, H = Histidine, I = Isoleucine, K = Lysine, L = Leucine, M = Methionine, N = Asparagine, P = Proline, Q = Glutamine, R = Arginine, S = Serine, T = Threonine, V = Valine, W = Tryptophan, Y = Tyrosine

For the L segment the substitutions at codon 277 (N277S, N277D) and codon 278 (S278N) were observed across many outbreaks. Codon 277 was identified as being under positive selection in human sequences from South Africa, Senegal and Angola. Whereas codon 278 occurred broadly in human, livestock, wildlife and vector isolates in outbreaks from East, West and Southern Africa (1944 to 2022).

For the M segment at codon 442, two nonsynonymous changes were detected (I442S and I442V). I442S appeared only in human isolates from Kenya (2018), while I442V appeared in both human and livestock sequences from Madagascar, Mauritania, South Africa and Zimbabwe during outbreaks in the 1970s–1980s. Whereas codon 659 (V659A) was widespread, occurring in all host categories across Angola, Burkina Faso, Kenya, Madagascar, Mauritania, Senegal, South Africa, Uganda, and Zimbabwe from the 1950s to 2020s, though it was detected under positive selection only in non-human sequences. For the S segment, the mutation N133S at codon 133 was detected in livestock and vector isolates sampled from Kenya, South Africa and Zimbabwe between the 1960s and 2020s. Evidence consistent with diversifying selection at this codon was detected only in human-derived sequences, as supported by FUBAR analysis.

### Spatiotemporal distribution of RVFV adaptive mutations in Africa

**Fig. 6:**
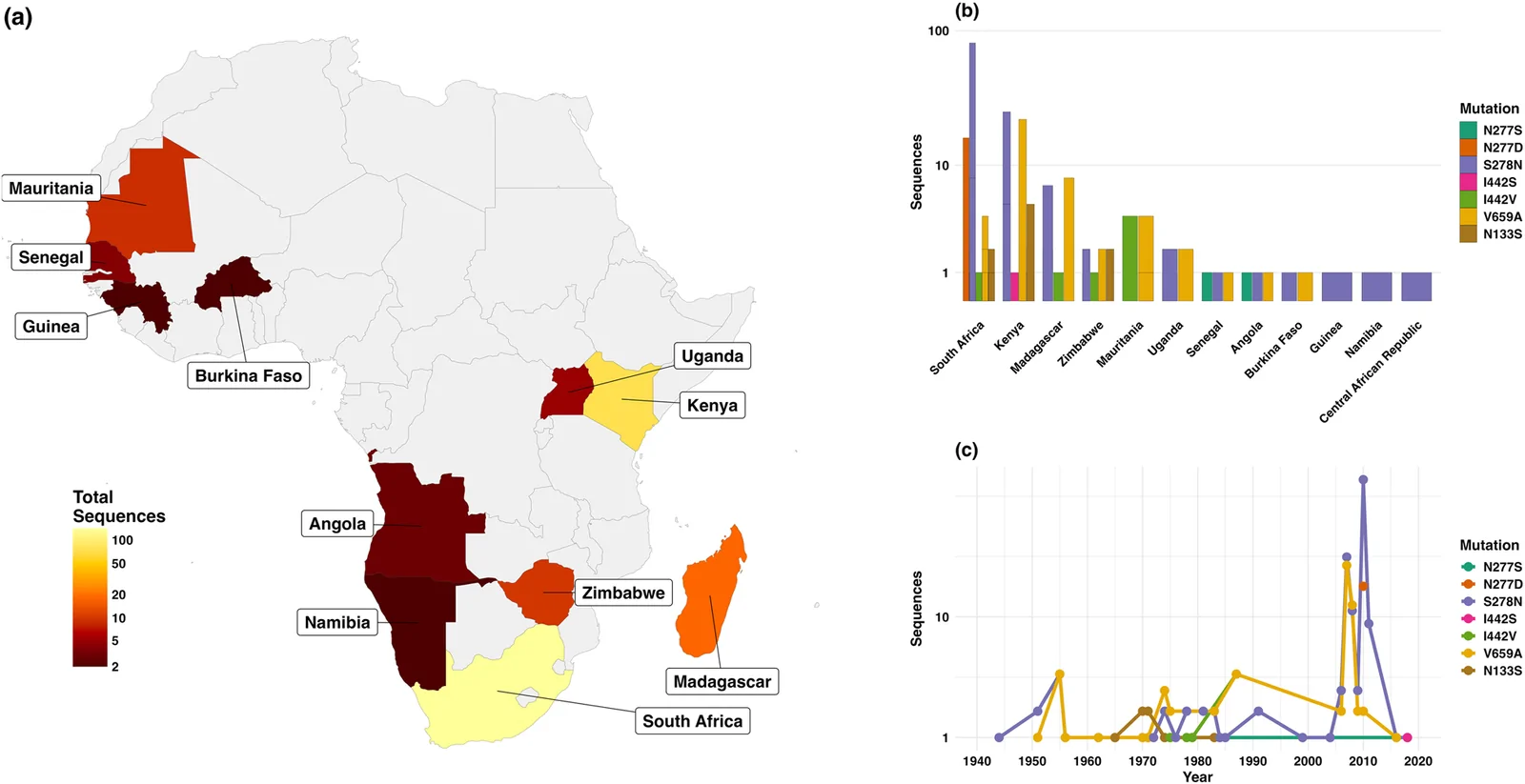
Spatiotemporal distribution of RVFV adaptive mutations in Africa. **a** A map of Africa showing countries with the total number of sequences containing at least one adaptive mutation. The color intensity represents the number of sequences per country. **b** A bar plot showing the number of sequences carrying each adaptive mutation by country, allowing comparison of mutation occurrence across geographic locations. **c** Temporal distribution of adaptive RVFV mutations across time

Several countries across Africa showed evidence of the above-mentioned mutations associated with viral evolution and host adaptation (Fig. 6). South Africa, Kenya and Madagascar carried the highest number of sequences with these mutations, followed by Mauritania, Zimbabwe and Uganda. Low numbers were detected in Namibia, Guinea, Burkina Faso and Angola as shown in Fig. 6a. The L segment mutations N277D, N277S and S278N were widely distributed with S278N showing the broadest range across East, West and Southern Africa (Fig. 6b). The M segment mutations I442S and I442V were more geographically restricted with I442S occurring only in Kenya in the 2018 epidemic, while I442V was confined to historical outbreaks in Madagascar, Mauritania, South Africa and Zimbabwe (1970s–1980s). The M codon V659A was the most geographically widespread, present across almost all countries represented in the dataset. The S segment mutation N133S was observed in Kenya, South Africa and Zimbabwe. Temporal trends showed that S278N persisted over the longest timeframe, ranging from earlier isolates to recent epidemics. I442V was restricted to the 1970s–1980s. V659A was recurrently observed across multiple decades and geographic regions, while N133S appeared between the 1960s and 2020s (Fig. 6c).

### Selection pressure analyses across hosts and genome segments

The analyses done across the L, M and S segments revealed that RVFV evolution is predominantly shaped by purifying selection, which is consistent with the high functional constraint of essential viral proteins. However, five codon sites, corresponding to seven recurrent amino acid mutations showed evidence of diversifying selection across human and non-human hosts during outbreaks in Africa (Table 2).

**Table 2:** Codon sites showing evidence of diversifying selection in RVFV across hosts

Host category	Segment	Sites	Model	dN/dS(β/α)	Posterior probability	Selection pressure
Human	L	277	FUBAR	14.657/0.793	0.9834	Diversifying
Human	L	278	FUBAR	9.771/0.828	0.9405	Diversifying
Human	M	442	FUBAR	7.372/0.758	0.9543	Diversifying
Human	S	133	FUBAR	4.657/0.736	0.9038	Diversifying
Non-human	M	659	FUBAR	5.374/0.698	0.9367	Diversifying

From the human sequences, four codon sites showed FUBAR-supported evidence consistent with diversifying selection (Table 2). Within the L segment, codon 277 (N277D/S) showed strong support (posterior probability = 0.9834; dN/dS ratio of 14.657/0.793), while codon 278 (S278N) showed moderate support (posterior = 0.9405; dN/dS = 9.771/0.828). In the M segment, codon 442 (I442S/V) also showed moderate support (posterior = 0.9543; dN/dS = 7.372/0.758). In the S segment, codon 133 (N133S; NSs protein) showed moderate support (posterior = 0.9038; dN/dS = 4.657/0.736). No codon sites met the predefined thresholds as detected by SLAC and FEL models.

Among the non-human isolates, only one codon site showed FUBAR-supported evidence consistent with diversifying selection. Codon 659 (V659A) in the M segment showed moderate support (posterior = 0.9367 with dN/dS = 5.374/0.698). No codon sites were detected in the L or S segments of livestock, vector or wildlife-derived sequences.

## Discussion

This study provides continent-wide insight into RVFV genomic evolution by integrating geographic, temporal, host and segment-specific analyses across outbreaks reported in 13 African countries between 1944–2022. The identification of 7,339 protein-coding mutations across 596 RVFV segment sequences analyzed in this study highlights substantial viral diversity across the continent. Within this broader mutational landscape, seven recurrent nonsynonymous substitutions were detected in functionally important viral proteins, identifying candidate hotspots of adaptive significance. These substitutions are potentially linked to enhanced replication efficiency, immune evasion or host adaptation. Collectively, these findings (Table 1) provide new insights into how RVFV adapts its genome during transmission across human, livestock, vectors and wildlife populations.

The findings show that RVFV evolution in Africa is geographically and temporally heterogeneous. South Africa, Madagascar and Kenya show recurrent episodes of high diversity, while Egypt and Central Africa exhibited more conserved viral populations. Notably, Mauritania showed the highest mutation burden during the 1987 outbreak, possibly reflecting a single but intense episode of viral diversification while Angola and Senegal showed elevated but sporadic mutation counts which is consistent with localized transmission events captured by limited genomic sampling. These spatiotemporal differences are further supported by the stepwise changes observed between successive sampled years in countries with denser genomic data, highlighting that temporal fluctuations reflect transitions between available sampling points rather than continuous year-to- year surveillance [64].

Kenya and Madagascar also displayed wide interquartile ranges and outliers (Fig. 2), consistent with repeated introductions or sustained circulation as well as climatic conditions that enable RVFV persistence. These findings are consistent with earlier reports of lineage C diversification and multiple subclades circulating in Kenya during the 2006 −2007 epidemic [33] and Uganda [13]. However, the reduced genetic diversity observed in North Africa may stem from ecological constraints due to limited competent *Aedes* and *Culex* vector populations [65]. Limited genomic sampling from previous RVFV outbreaks, for example only 16 sequences from Egypt are available in the NCBI virus repository, substantially restricting the ability to capture the true extent of viral diversity and evolutionary dynamics in this region.

Comparative analysis across host categories showed marked differences across genome segments. Overall, the M segment exhibited lower diversity than the L and S segments, consistent with the functional constraints of the envelope glycoproteins Gn and Gc encoded by the medium (M) segment [5], which may evolve more slowly due to structural and immune requirements. The reduced diversity in human genomes likely reflects transmission bottlenecks at spillover, where only a narrow subset of the viral diversity that is circulating in livestock and vectors is transmitted to humans, often resulting in short infection chains. Phylogenetic clustering of the M segment illustrates in Fig. 4, the impact of host-associated genetic bottlenecks [66]. With the human isolates clustering in narrow sub-clades embedded within livestock and vector-derived clades, this suggests that humans capture only a limited subset of the circulating viral diversity during transmission [67]. Whereas animal reservoirs retain broad lineage diversity across decades, demonstrating their role as long-term reservoirs. These genetic bottlenecks may allow fixation of beneficial mutations that enhance viral fitness and immune evasion in the new hosts. Such evolutionary patterns have been observed in other arboviruses such as Zika virus [68] and West Nile virus [69], where reservoirs retain broad genomic diversity and occasionally seed spillover lineages in humans.

Given that the M segment encodes envelope glycoproteins critical for host-cell interaction and viral entry, it was selected as the representative segment for ancestral host-state reconstruction to better understand the evolutionary drivers of cross-species transmission. The ancestral host state reconstruction and host transition network provided further insights into how RVFV circulates and spills over into humans across Africa. The observed high numbers in livestock-to-human transitions show the role of domestic ruminants as the primary amplifying hosts driving RVFV spillover into human populations and the principal source of onward spread [70]. The absence of wildlife to human transitions likely reflects limited genomic sampling from reservoirs or lower transmission efficiency from wildlife [71]. The tight human-only cluster observed around 2008–2010 (Fig. 4) represents a group of closely related human-derived sequences sampled within a narrow temporal window. However, distinguishing repeated spillovers from brief onward transmission would require denser human sampling and accompanying exposure. Together the phylogeny and ancestral state reconstruction analysis show how RVFV spillover into humans emerges. These findings show that livestock infections are a major driver of human RVFV outbreaks [70].

Host-associated differences in mutation frequency were also evident across RVFV genome segments [72]. The L and M segments showed significantly higher mutation counts in non-human sequences, which is likely associated with broader circulation across livestock, wildlife and vector transmission cycles where viral diversification may accumulate over time [11]. In contrast, the S segment showed significantly higher mutation counts in human-derived sequences, suggesting segment specific evolutionary dynamics that may reflect selective pressures, sampling structure and viral adaptation associated with spillover infections [73].

The interpretation of these candidate adaptive codons, should, however be considered in light of methodological differences among site-level selection models. The codon sites highlighted in this study were supported by FUBAR, whereas no positively selected sites were detected by SLAC or FEL under the thresholds applied. This difference likely reflects methodological variation among site-level selection approaches. FUBAR is a Bayesian method with greater sensitivity for detecting pervasive but relatively weak diversifying selection across phylogenies [54], whereas SLAC and FEL are generally more conservative and may have lower power when sequence divergence is limited or when substitutions are sparse [74]. The identified codons may therefore be interpreted as putative adaptive sites showing signatures consistent with diversifying selection.

Previous phylogenetic and molecular studies have consistently shown that RVFV evolution is largely constrained by purifying selection, preserving the functional integrity of viral proteins across outbreaks. For example, a study by Bird et al., in 2007 showed minimal amino acid divergence on the polymerase and glycoproteins of RVFV among African isolates [6]. Subsequent studies from Mauritania, Senegal, South Africa and Kenya also identified few non-synonymous changes and low dN/dS ratios, showing negative selection acts to preserve essential replication and virulence. During these outbreaks, most amino acid substitutions were conservative, showing evolutionary stability rather than rapid adaptation.

In contrast to these past studies, this continent-wide analysis identified FUBAR-detected signatures consistent with diversifying selection at several codon sites, suggesting possible host-associated adaptive pressures [75]. FUBAR-supported codons in the L polymerase (277, 278), M glycoprotein (442) and the S NSs protein (133) from human-derived sequences, together with codon 659 in the M segment of non-human hosts, map to proteins associated with viral replication, immune recognition and host adaptation [7]. Signals in the L segment RNA polymerase may reflect adaptation favoring efficient replication in mammalian hosts, potentially influencing synthesis and replication speed, traits closely related to viral fitness [72]. Similarly, signals consistent with diversifying selection at codons 442 and 659 within the glycoproteins may alter envelope conformation and modulate antigenic sites, enhancing immune escape and receptor affinity [5]. These adaptive substitutions can increase viral binding efficiency to host cell receptors, while also maintaining structural stability of the virion [5]. Notably, the mutation at codon 133 in the NSs gene is particularly relevant given that the NSs acts as a major virulence factor known to antagonize interferon responses [6]. The N133S substitution was also reported in a recent study by Bakamutumaho et al., 2025, during investigations of hyperendemic RVFV in Southwestern Uganda [13]. Although it was described only as a recurrent substitution. In contrast, this present study, suggests that codon 133 of the S segment may be under diversifying selection, suggestive of ongoing molecular adaptation driven by human immune pressures. Such selection may enhance the virus’s ability to sustain replication under interferon-stimulated conditions [7].

Together, these findings extend our current understanding by showing that although RVFV evolution is largely constrained by purifying selection to maintain core functions, diversifying selection signals were identified at several candidate residues which may enhance RVFV fitness and host adaptation during outbreaks in Africa. These adaptive mutations could modify polymerase or glycoprotein changes, specifically those in the glycoproteins which could shift immune recognition by masking neutralizing epitopes or altering glycosylation motifs [60]. In turn, the immune-driven adaptations enhance viral persistence within endemic hosts while enabling occasional escape from preexisting herd immunity in livestock populations [76].

The coexistence of conserved and variable regions across RVFV segments also suggests a tradeoff between replication fidelity and immune recognition [77]. The conserved polymerase motifs ensure replication efficiency, while the variable glycoprotein loops allow evasion of neutralizing antibodies [78]. This kind of adaptive trade-off represents a hallmark of RNA virus evolution, allowing RVFV to balance fitness and immune escape [78]. From a one health perspective, the findings from this study underscore that viral adaptation in animal reservoirs underpins the risk of outbreaks in human. The continuous replication in livestock and mosquitoes allows the accumulation of fitness enhancing mutations, which can ultimately be transmitted to humans where immune pressures drive further refinement of adaptive traits [62].

Understanding RVFV adaptation has clear practical applications. For example, the relative genetic stability of the medium (M) segment glycoproteins supports their role as vaccine targets, although their exposure to immune-driven selection necessitates inclusion of conserved epitopes to ensure long-term vaccine efficacy [27]. Historical vaccines such as Smithburn, MP-12 and Clone-13 primarily relied on inactivation and attenuation rather than targeting the evolutionarily stable genomic regions [79]. Integrating genomic selection data can improve antigen design through prioritization of conserved and functionally constrained residues which are less prone to adaptive evolution [24].

In this context, immunoinformatics approaches have emerged as powerful tools to bridge genomic insights and vaccine designs. Recent research studies have applied these methods to design epitopes for use in ruminants demonstrating the feasibility of combining both B-cell and T-cell epitopes to elicit broad protective responses [76]. These findings complement such efforts by identifying candidate adaptive codon sites that can be considered when selecting epitopes, as these residues may compromise vaccine coverage through immune escape [5]. By integrating molecular evolution, immunoinformatics and computational modelling for immunogen selection, future vaccine design strategies could yield broadly protective immunogens while minimizing antigenic drift [24].

Despite these insights, several limitations warrant consideration. The geographic and temporal distribution of available RVFV genomes was uneven, with some countries and outbreak periods underrepresented [60]. This is because the publicly available genomic data were disproportionately contributed by a limited number of countries particularly South Africa (*n* = 321) and Kenya (*n* = 136) which together accounted for 76.7% of all sequences analyzed. Contributions were substantially smaller from Madagascar (*n* = 33), Zimbabwe (*n* = 27), Egypt (*n* = 16) and the Central African Republic (*n* = 16), while several other affected countries were represented by fewer than 10 sequences. Because genomic surveillance intensifies during epidemics or epizootics and declines thereafter, the observed spatial and temporal diversity patterns may partly reflect episodic sequencing activity rather than continuous endemic transmission [59]. The country-level mutation frequency estimates may also be influenced by localized sampling practices that overrepresent specific outbreak sites rather than the national viral genetic diversity [73]. This could have potentially affected estimates of mutation frequencies and the detection of selection pressure. Incorporating structural modelling and molecular docking analyses could provide insights into how these adaptive mutations influence protein conformation, receptor binding and antibody neutralization [76]. Such multidisciplinary efforts will strengthen genomic to phenotypic inference and accelerate vaccine development efforts against RVFV and other emerging or re-emerging viruses of epidemic and pandemic potential.

## Conclusion

This study provides continent-wide insight into the evolutionary landscape of RVFV across multiple outbreaks in Africa between 1944 and 2022. RVFV genetic diversity was geographically and temporally heterogeneous, and livestock-associated transmission appears to be a major source of human spillover. While purifying selection remained the dominant evolutionary force, a limited number of codon sites showed putative adaptive signals consistent with host-associated pressures. These findings support genomic surveillance, vaccine target prioritization and One Health outbreak preparedness in Africa.

## Supplementary Information

Additional file 1: Figure S1. Root-to-tip regression analysis showing temporal signal in Rift Valley fever virus M segment sequences.

Additional file 2: Figure S2. Phylogenetic structure of Rift Valley fever virus L segment by host and geographic region.

Additional file 3: Figure S3. Phylogenetic structure of Rift Valley fever virus S segment by host and geographic region.

Additional file 4: Dataset S4. The (Combined-Mutation.csv) dataset contains the complete list of 7,339 protein-coding Rift Valley fever virus mutations identified from the 596 complete RVFV segment sequences across human and non-human outbreaks in Africa (1944—2022). The mutations were identified across the L, M and S segments sequences. This associated metadata also includes accession numbers, country of origin, host, lineage assignment, sampling years, affected protein, codon position and observed amino acid substitutions.

Additional file 5: Dataset S5. The initial metadata (metadata.csv) which contained the initial merged metadata for human and non-human for RVFV M segment sequences prepared for phylogenetic inference, temporal signal analyses and lineage distribution and root-to-tip regression analysis in the M segment.

Additional file 6: Dataset S6. The retained filtered metadata (metadata.filtered.csv) for RVFV M segment sequences retained in the final phylogenetic analysis. This is the file after FASTA deduplication and sequence processing and was used for multiple sequence alignment, maximum likelihood phylogenetic inference and temporal signal analysis.

Additional file 7: Dataset S7. Host-state transition matrix inferred from the RVFV M segment phylogeny. The file contains directed host-to-host transition counts reconstructed from ancestral host-state analysis under an equal-rates Markov model. Rows summarize inferred transitions between human, livestock, wildlife and vector host categories, including within host transitions and spillover events into humans.

## Data Availability

Genomic data analyzed in this study were retrieved from the NCBI virus database under the taxonomy identifier: Rift Valley fever virus (taxid:11588). The accession numbers of all RVFV sequences included in this study are provided in Additional file 4 (Dataset S4). The query used to retrieve the sequences is available at https://www.ncbi.nlm.nih.gov/labs/virus/vssi/#/virus?SeqType_s=Nucleotide&VirusLineage_ss=Rift%20Valley%20fever%20virus,%20taxid:11588&Completeness_s=complete&Region_s=Africa&VacStrain_s=exclude&LabHost_s=exclude. The workflow, custom scripts and processed datasets generated from this study are available on GitHub https://github.com/OmaraI/rvfv-evolution-africa.
