# Supplementary material for: Evolutionary dynamics and molecular adaptation of Rift Valley fever virus across human and non-human outbreaks in Africa
Source: BMC Genomics. 2026 Jun 15;27:700. doi: 10.1186/s12864-026-13002-4 (PMC13495268; doi:10.1186/s12864-026-13002-4)
Supplement: Supplementary file 1 [file 12864_2026_13002_MOESM1_ESM.docx]

**Figure S1:** Root-to-tip regression analysis of RVFV M segment genomes showing the relationship between genetic divergence and sampling year. A positive correlation was observed (β = 1.88 × 10⁻⁴ substitutions/site/year), supporting measurable temporal signal and clock-like evolution within the dataset. Temporal outliers were filtered using the TreeTime --clock-filter 3 option prior to final time-scaled phylogenetic reconstruction.
