# Supplementary material for: Evolutionary dynamics and molecular adaptation of Rift Valley fever virus across human and non-human outbreaks in Africa
Source: BMC Genomics. 2026 Jun 15;27:700. doi: 10.1186/s12864-026-13002-4 (PMC13495268; doi:10.1186/s12864-026-13002-4)
Supplement: Supplementary file 2 [file 12864_2026_13002_MOESM2_ESM.docx]

**Figure S2:** **Phylogenetic structure of the RVFV L segment by host and geographic region.** The tips are colored by host category (Human, Livestock, Wildlife and Vector). The heatmaps show the geographic regions (North Africa: Egypt, East: Uganda, Kenya, Madagascar, Southern Africa: South Africa, Namibia, Zimbabwe, Central Africa: Central African Republic, Angola and West Africa: Mauritania, Senegal, Burkina Faso, Guinea). Bootstrap values are shown on major nodes to indicate statistical support (≥70%).
